# Ultrastructure and molecular phylogeny of *Calkinsia aureus*: cellular identity of a novel clade of deep-sea euglenozoans with epibiotic bacteria

**DOI:** 10.1186/1471-2180-9-16

**Published:** 2009-01-27

**Authors:** Naoji Yubuki, Virginia P Edgcomb, Joan M Bernhard, Brian S Leander

**Affiliations:** 1 Canadian Institute for Advanced Research, Program in Integrated Microbial Biodiversity, Departments of Botany and Zoology, University of British Columbia, 6270 University Boulevard, Vancouver, BC V6T 1Z4, Canada; 2 Department of Geology and Geophysics, Woods Hole Oceanographic Institution, Woods Hole, MA 02543, USA

## Abstract

**Background:**

The Euglenozoa is a large group of eukaryotic flagellates with diverse modes of nutrition. The group consists of three main subclades – euglenids, kinetoplastids and diplonemids – that have been confirmed with both molecular phylogenetic analyses and a combination of shared ultrastructural characteristics. Several poorly understood lineages of putative euglenozoans live in anoxic environments, such as *Calkinsia aureus*, and have yet to be characterized at the molecular and ultrastructural levels. Improved understanding of these lineages is expected to shed considerable light onto the ultrastructure of prokaryote-eukaryote symbioses and the associated cellular innovations found within the Euglenozoa and beyond.

**Results:**

We collected *Calkinsia aureus *from core samples taken from the low-oxygen seafloor of the Santa Barbara Basin (580 – 592 m depth), California. These biflagellates were distinctively orange in color and covered with a dense array of elongated epibiotic bacteria. Serial TEM sections through individually prepared cells demonstrated that *C. aureus *shares derived ultrastructural features with other members of the Euglenozoa (e.g. the same paraxonemal rods, microtubular root system and extrusomes). However, *C. aureus *also possessed several novel ultrastructural systems, such as modified mitochondria (i.e. hydrogenosome-like), an "extrusomal pocket", a highly organized extracellular matrix beneath epibiotic bacteria and a complex flagellar transition zone. Molecular phylogenies inferred from SSU rDNA sequences demonstrated that *C. aureus *grouped strongly within the Euglenozoa and with several environmental sequences taken from low-oxygen sediments in various locations around the world.

**Conclusion:**

*Calkinsia aureus *possesses all of the synapomorphies for the Euglenozoa, but lacks traits that are specific to any of the three previously recognized euglenozoan subgroups. Molecular phylogenetic analyses of *C. aureus *demonstrate that this lineage is a member of a novel euglenozoan subclade consisting of uncharacterized cells living in low-oxygen environments. Our ultrastructural description of *C. aureus *establishes the cellular identity of a fourth group of euglenozoans, referred to as the "Symbiontida".

## Background

The Euglenozoa is a clade of eukaryotic microorganisms with very diverse lifestyles and that tentatively falls within one of six emerging supergroups of eukaryotes, namely the "Excavata" [1-3]. Most euglenozoans cluster within three major subgroups that have been established with both molecular phylogenetic analyses and combination of ultrastructural characteristics (e.g. the same tripartite flagellar root system): the Kinetoplastida, the Euglenida and the Diplonemida [3-8]. Kinetoplastids possess mitochondria with a uniquely structured genome, called "kinetoplast" DNA, and the group includes both free-living phagotrophic lineages (e.g. bodonids) and parasitic lineages (e.g. trypanosomatids such as *Trypanosoma *and *Lieshmania*). Euglenids possess a cytoskeleton, or "pellicle", consisting of overlapping proteinaceous strips that are arranged either longitudinally or helically, and the group includes bacteriovorous lineages (e. g. *Petalomonas*), eukaryovorous lineages (e.g. *Peranema*), osmotrophic lineages (e.g. *Menodinium*) and photosynthetic lineages (e.g. *Euglena*). The mitochondria of kinetoplastids and euglenids possess cristae that are distinctively discoidal in shape. By contrast, diplonemids consist of only two genera, *Diplonema *and *Rhynchopus*, with sack-shaped cells, short flagella and flattened mitochondrial cristae and without kinetoplast DNA, pellicle strips, and paraxonemal rods.

Ultrastructural studies have also demonstrated lineages of euglenozoans that do not fall neatly within any of the three established subgroups, such as *Postgaardi mariagerensis*, which inhabits low oxygen environments and is covered with epibiotic bacteria [9]. Currently, *P. mariagerensis *is grouped together with another poorly understood anoxic flagellate, namely *Calkinsia aureus*, as incertae sedis within the Euglenozoa [3]; although molecular data is unavailable for both species, one author has chosen to classify them within a taxon called the "Postgaardea" [10,11]. *C. aureus *was originally collected from anoxic sediments near Woods Hole, MA (USA) and described with only line drawings as a member of the euglenid family Petalomonidae; this conclusion was based on the appearance of a rigid cell containing strip-like surface striations [12]. However, *C. aureus *was subsequently collected from low-oxygen sediments in the Santa Barbara Basin, CA (USA) and partially studied with light and scanning electron microscopy (LM and SEM, respectively) [13,14]. These studies demonstrated that like *P. mariagerensis*, *C. aureus *was covered with the rod-shape epibiotic bacteria, rather than pellicle strips per se.

The ultrastructure and molecular phylogenetic position of *C. aureus *is currently unknown. These data are expected to help establish robust inferences about the overall diversity of euglenozoans and the ultrastructure of prokaryote-eukaryote symbioses within the group and beyond. The main goals of this study were to characterize the ultrastructure and molecular phylogenetic position of *C. aureus *using small subunit (SSU) rDNA sequences and transmission electron microscopy (TEM) of serially sectioned cells. Our results demonstrated that *C. aureus *is the first member of a novel group of anoxic euglenozoans – referred to here as the "Symbiontida" – to be characterized at both the molecular and ultrastructural levels. A companion study centered on the molecular identity and detailed ultrastructure of the epibiotic bacteria on *C. aureus *is currently underway.

## Methods

### Collection of organisms

*Calkinsia aureus *was collected using a Soutar box corer or MC-800 multi corer from the sea floor sediment (580 – 592 m in depth) of the Santa Barbara Basin, California, USA in September of 2007 and June of 2008. Sediment core samples were collected on the R/V *Robert Gordon Sproul*. Some sediment samples were immediately fixed for transmission electron microscopy (TEM) with an equal volume of 4% (v/v) glutaraldehyde in 0.2 M sodium cacodylate buffer (SCB) (pH 7.2) and stored at 4°C. The remaining sediment samples were stored in 50 ml plastic tubes at 4°C and subsequently processed for light microscopy, scanning electron microscopy (SEM) and DNA extraction.

### Light and electron microscopy

Light micrographs of over 100 living cells were taken using a Zeiss Axioplan 2 imaging microscope and a Leica DC500 digital chilled CCD camera.

Cells of *C. aureus *were prepared for SEM by mixing an equal volume of fixative solution containing 4% (v/v) glutaraldehyde in 0.2 M SCB (pH 7.2) at room temperature. The fixed cells were mounted on polycarbonate Millipore filters (13-mm diam., 5-μm pore size) or glass plates coated with poly-L-lysine at room temperature for 1 hr. The cells were rinsed with 0.1 M SCB and fixed in 1% osmium tetroxide for 30 min. The osmium-fixed cells were then rinsed with 0.1 M SCB and dehydrated with a graded ethanol series from 30% to absolute ethanol before being critical point dried with CO_2 _using a Tousimis Critical Point Dryer. The dried cells were then coated with gold using a Cressington 208HR High Resolution Sputter Coater, and observed with a Hitachi S-4700 field emission scanning electron microscope.

Cells of *C. aureus *prepared for TEM were kept in fixative solution for two months before being individually isolated from the surrounding sediment in the sample. Isolated cells were rinsed with 0.2 M SCB (pH 7.2) three times and then fixed in 1% (w/v) osmium tetroxide in 0.2 M SCB (pH 7.2) at room temperature for 1 hr before being dehydrated through a graded series of ethanol and 100% acetone. The dehydrated cells were then infiltrated with acetone-Epon 812 resin mixtures and 100% resin. Individual cells were flat embedded and serial sectioned in different orientations (i.e. transverse and longitudinal). Ultra-thin serial sections were collected on copper, Formvar-coated slot grids and stained with 2% (w/v) uranyl acetate and lead citrate [15] before being observed using a Hitachi H7600 electron microscope. A total of 899 micrographs from 12 different cells were observed.

Two different media were used in an attempt to culture *C. aureus*: 5% of TYGM-9 (ATCC medium 1171) and 5% of modified PYNFH medium (ATCC medium 1134), diluted in anoxic and axenic seawater at 4°C. However, the cells did not grow in either medium.

### DNA extraction, PCR amplification, alignment and phylogenetic analysis

Twenty individual cells of *C. aureus *that corresponded to the original species description (distinctive color, size, shape and motility [12]) were isolated from the sediment and washed twice in sterilized seawater. Because of the highly distinctive morphology of *C. aureus *and the precautions taken, the possibility of contamination is exceedingly low. Genomic DNA was extracted from the cells using MasterPure Complete DNA and RNA purification Kit (Epicentre, WI, USA). The polymerase chain reaction (PCR) was performed using a total volume of 25 μl and the PuRe Taq Ready-To-Go PCR beads kit (GE Healthcare, Buckinghamshire, UK). Nearly the entire SSU rRNA gene was amplified from genomic DNA using eukaryotic universal primers (PF1: 5'-GCGCTACCTGGTTGATCCTGCCAGT-3' and R4: 5'-GATCCTTCTGCAGGTTCACCTAC-3'). The PCR protocol had an initial denaturation stage at 95°C for 2 min; 35 cycles involving 94°C for 45 s (denaturation), 55°C for 45 s (annealing), and 72°C for 1.5 min (extension); and final extension at 72°C for 5 min. The amplified DNA fragments were purified from agarose gels using UltraClean 15 DNA Purification Kit (MO Bio, CA, USA), and then cloned into the TOPO TA Cloning Kit (Invitrogen, CA, USA). The *C. aureus *sequence was deposited in DDBJ/EMBL/GenBank under the accession number EU753419.

The SSU rRNA sequence of *C. aureus *was visually aligned with taxa representing all of the major groups of eukaryotes, forming (i) a 38-taxon alignment with ambiguously aligned regions excluded (988 unambiguously aligned positions). In order to more comprehensively evaluate the phylogenetic position of *C. aureus *within the Euglenozoa, we analyzed three additional datasets: (ii) a 35-taxon alignment of euglenozoan sequences and ten relatively short environmental sequences (760 unambiguously aligned positions); (iii) a 29-taxon alignment of euglenozoan sequences including three fast-evolving euglenid sequences – namely *Astasia torta *(AF403152), *Menoidium bibacillatum *(AF247598) and *Ploeotia costata *(AF525486) – and excluding the short environmental sequences (734 unambiguously aligned positions); and (iv) a 25-taxon alignment of euglenozoan sequences excluding both the short environmental sequences and the fastest-evolving euglenid sequences (1025 unambiguously aligned positions). The highly divergent sequences from phagotrophic euglenids produced a large number of ambiguously aligned regions in the 35-taxon and 29-taxon alignments; accordingly, these regions were excluded from our analyses.

PhyML [16] was used to analyze all four datasets (one heuristic search per dataset) with maximum-likelihood (ML) using a general-time reversible (GTR) model of base substitutions [17] that incorporated invariable sites and a discrete gamma distribution (eight categories) (GTR + I + G model). The GTR model was selected using the program MrAIC 1.4.3 with PhyML http://www.abc.se/~nylander/mraic/mraic.html. Model parameters were estimated from each of the original datasets. ML bootstrap analysis was conducted with the same settings described above (100 pseudoreplicates; one heuristic search per pseudoreplicate).

The four alignments were also analyzed with Bayesian methods using the MrBayes program [18]. The program was set to operate with a gamma distribution and four Monte-Carlo-Markov chains (MCMC) starting from a random tree. A total of 2,000,000 generations were calculated with trees sampled every 50 generations and with a prior burn-in of 100,000 generations (2000 sampled trees were discarded; burn-in was checked manually). A majority rule consensus tree was constructed from 38,000 post-burn-in trees. Posterior probabilities correspond to the frequency at which a given node was found in the post-burn-in trees. Independent Bayesian runs on each alignment yielded the same results.

### Archiving

A digital archive of this paper is available from PubMed Central and print copies are available from libraries in the following five museums: Natural History Museum Library (Cromwell Road, London, SW7 5BD, UK), American Museum of Natural History (Department of Library Services, Central Park West at 79th St., New York, NY, 10024, USA), Muséum national d'Histoire naturelle (Direction des bibliothèques et de la documentation, 38 rue Geoffroy Saint-Hilaire, 75005 Paris, France), Russian Academy of Sciences (Library for Natural Sciences of the RAS Znamenka str., 11, Moscow, Russia) and Academia Sinica (Life Science Library, 128 Sec. 2 Academia Rd, Nankang, Taipei 115, Taiwan R.O.C.).

## Results

### General Morphology

**Figure 1:**
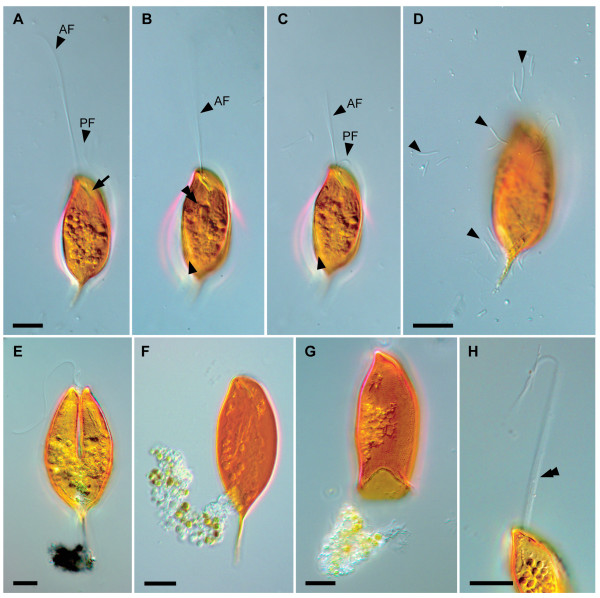
**Differential interference contrast images of the living cell of *Calkinsia aureus***. The micrographs show the distinctively orange color of the cell, two flagella, epibiotic bacteria and ingested material. **A**. Anterior flagellum (AF) and the posterior flagellum (PF) inserted into an anterior opening (arrow). The ingested material is present in the middle and posterior regions of the cell.** B**. Surface striations (arrowhead) and a longitudinal rod-like structure (double arrowhead) indicative of a feeding apparatus. **C**. AF and PF emerging from the anterior opening. The arrowhead shows striation on the surface of the cell. **D**. Bacteria (arrowheads) that have disassociated with *C. aureus*. **E**. A cell undergoing division showing a longitudinal cleavage furrow starts from the anterior end. The ingested material is present in the middle and posterior regions of the cell.** F**. Clear cytoplasm extruded from posterior of the cell. **G**. Bright orange extracellular matrix. **H**. Bundle of extrusomes (double arrowhead) that have been discharged from extrusomal pocket through the anterior opening. (bars = 10 μm, **A-C **at same scale).

**Figure 2:**
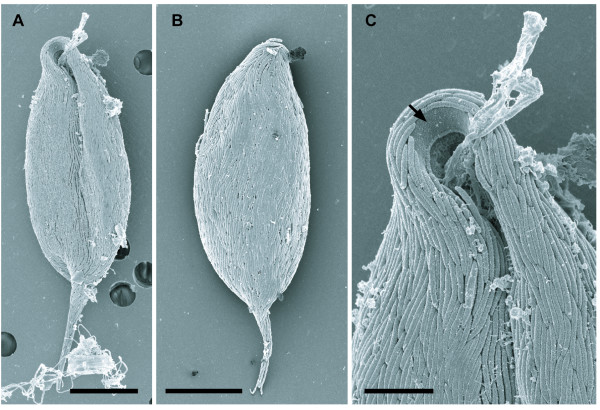
**Scanning electron micrographs (SEM) of *Calkinsia aureus***. **A**. The ventral side of *C. aureus *showing the anterior opening, a longitudinal groove and epibiotic bacteria. **B**. The dorsal side of the *C. aureus *showing the epibiotic bacteria. (**A, B **bars = 10 μm). **C**. High magnification SEM of the anterior vestibular opening showing the absence of epibiotic bacteria on the extracellular matrix (arrow). (bar = 3 μm).

**Figure 3:**
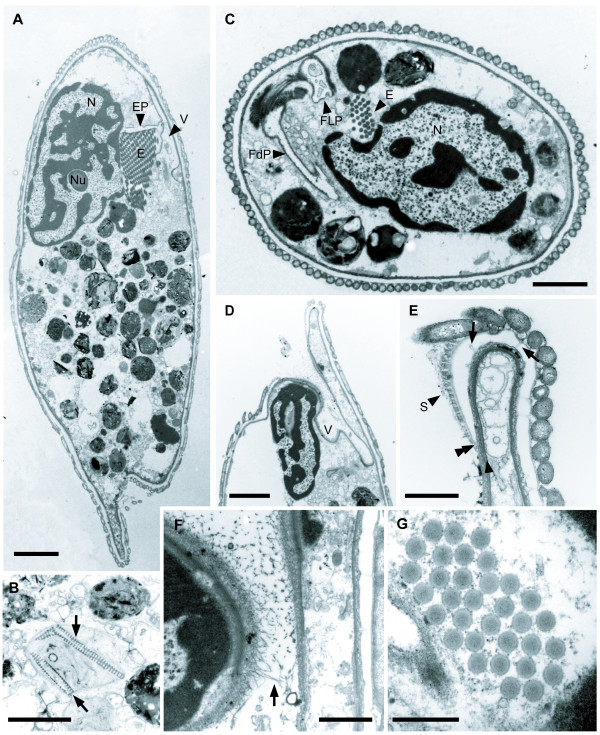
**Transmission electron micrographs (TEM) showing the general morphology of *Calkinsia aureus***. **A**. Sagittal TEM showing the nucleus (N) with condensed chromatin and a conspicuous nucleolus (Nu), a battery of extrusomes (E), the vestibulum (V) located on the dorsal side of the cell, ingested material and epibiotic bacteria on the extracellular matrix. The extrusomal pocket (EP) branched from the vestibulum (V) (bar = 4 μm). **B**. Ingested material containing diatom frustules (arrow). (bar = 2 μm). **C**. Cross section of the cell through the nucleus (N), the battery of extrusomes (E), the flagellar pocket (FLP) and the feeding pocket (FdP). (bar = 2 μm). **D**. High magnification view through the vestibulum (V) that is opened on the ventral side of the cell. **E**. High magnification view through the anterior opening showing the termination of the extracellular matrix (double arrowhead) and fine somatonemes (S) or hair-like structures on the perforated matrix (arrows) that is not covered with epibiotic bacteria. The arrowhead indicates the supportive microtubular sheet that lines the inside of the cytostome and turns along the cell surface. (**D**, **E**, bars = 1 μm). **F**. Hairs (arrow) on the wall of the vestibulum (V). (bar = 1 μm). **G**. Cross section showing the battery of tubular extrusomes (E). (bar = 2 μm).

*Calkinsia aureus *ranged from 41.7–71.2 μm long (average length = 56.7 μm, n = 32) and from 14.5–23.3 μm wide (average width = 18.3 μm, n = 32). The oval-shaped cells were distinctively orange in color, dorsoventrally compressed, and possessed a tapered tail that was about 10 μm long (Figure 1). Two heterodynamic flagella were inserted within a subapical depression at the anterior end of the cell. The longer anterior flagellum was about twice the length of the cell and was held straight forward during gliding. The shorter posterior flagellum was half the length of the cell and was usually positioned within a ventral groove. Colorless rod-shaped epibiotic bacteria were oriented along the longitudinal axis of the cell (Figures 1B–D, 2). The posterior half of the cell usually contained an accumulation of spherical food bodies, some of which contained diatom frustules (Figures 1A–F, 3A–B). Cyst formation and sexual reproduction were not observed. Asexual reproduction was achieved by cell division along the longitudinal axis of the cell. Following the replication of the flagellar apparatus, a cleavage furrow formed at the anterior end of the cell and advanced toward the posterior end of the cell (Figure 1E).

### Cell Surface and Extracellular Matrix

**Figure 4:**
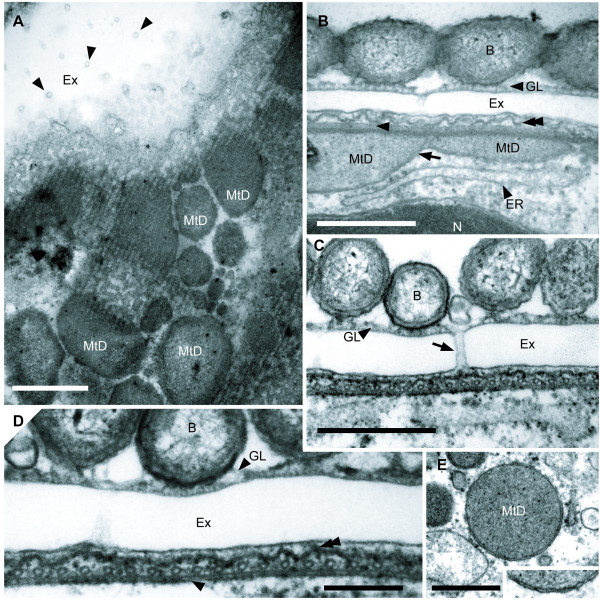
**Transmission electron micrographs (TEM) showing the surface ultrastructure of *Calkinsia aureus***. **A**. Tangential TEM section showing conduit-like perforations (arrowheads) embedded within the extracellular matrix (Ex), an array of microtubules, and mitochondrion-derived organelles (MtD). (bar = 1 μm). **B**. Mitochondrion-derived organelles (MtD) with two membranes (arrow) above the ER. The convoluted appearance of the cell plasma membrane (double arrowhead) and a longitudinal view of a microtubule (arrowhead) are also shown. A glycocalyx (GL) covers the surface of the extracellular matrix (Ex). **C**. Transverse TEM showing the epibiotic bacteria (B), the glycocalyx (GL), a conduit-like perforation (arrow) through the extracellular matrix (Ex) and the underlying sheet of microtubules (**B, C**, bars = 500 nm). **D**. High magnification view showing the epibiotic bacteria (B), the glycocalyx (GL), the extracellular matrix (Ex), the cell plasma membrane (double arrowhead), and the double-layered structure (arrowhead; derived from the dorsal lamina) beneath a sheet of inter-connected microtubules (bar = 200 nm). **E**. Mitochondrion-derived organelles (MtD) (bar = 500 nm). Inset: High magnification TEM showing the two membranes that surround the mitochondrion-derived organelles (width of inset = 400 nm).

**Figure 5:**
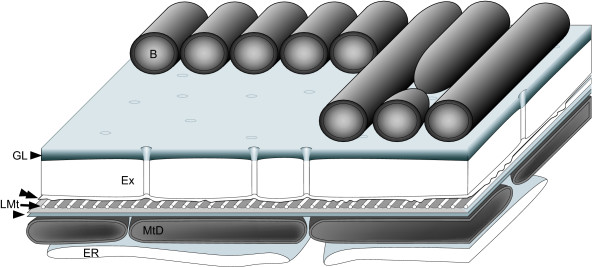
**Diagram of the cell surface of *Calkinsia aureus***. The diagram shows epibiotic bacteria (B), the glycocalyx (GL), the perforated extracellular matrix (Ex), the host cell plasma membrane (double arrowhead), the linked microtubules (LMt), the double-layered structure (arrowhead), mitochondrion-derived organelles (MtD) and cisternae of endoplasmic reticulum (ER).

The longitudinally arranged, epibiotic bacteria consisted of only one rod-shaped morphotype (3–5 μm long and 0.350 μm wide) that collectively formed a dense coat over the entire surface of the host cell (Figures 2, 3A, 3C). At least 128 epibiotic bacteria were observed in transverse sections through one cell of *C. aureus *(Figure 3C). The cell surface beneath the epibiotic bacteria consisted of seven main layers (from outside to inside): (1) a glycocalyx, (2) a highly organized extracellular matrix, (3) the host cell membrane, (4) an array of parallel microtubules, (5) a double-layered lamella, (6) superficially arranged mitochondrion-derived organelles and (7) cisternae of endoplasmic reticulum (ER) (Figures 3, 4, 5). The extracellular matrix surrounded the entire cell except for the inside lining of the vestibulum, which leads to the flagellar pocket and feeding pockets (Figures 2C, 3D–E). The portion of the extracellular matrix positioned just inside the opening of the vestibulum lacked epibiotic bacteria and consisted of fine hair-like structures, or somatonemes (Figure 3E). The extracellular matrix beneath the epibiotic bacteria was coated with a thin glycocalyx (Figures 4B–D, 5). The extracellular matrix itself was bright orange, approximately 100 nm thick and perforated with hollow tubes that joined the plasma membrane of the host with the glycocalyx beneath the epibiotic bacteria (Figures 1G, 4A–C, 5).

An array of evenly spaced microtubules was positioned immediately beneath the plasma membrane of the host (Figures 4A, 4C–D, 5). These microtubules were derived from the dorsal lamina (DL) of the flagellar apparatus (see description below). Each microtubule was connected to neighboring microtubules with electron dense "arms" (Figure 4D). A double-layered lamella was positioned between the layer of microtubules and a deeper layer of mitochondrion-derived organelles (Figures 4A–B, 4D). The mitochondrion-derived organelles were discoidal in shape, were bounded by two membranes and lacked mitochondrial cristae or inclusions such as kinetoplasts (Figures 4A–B, 4E). Moreover, we did not observe any evidence of euglenid-like pellicle features, such as the presence of S-shape proteinaceous strips or discontinuities in the layer of microtubules.

### Nucleus, Vestibulum and Associated Pockets

**Figure 6:**
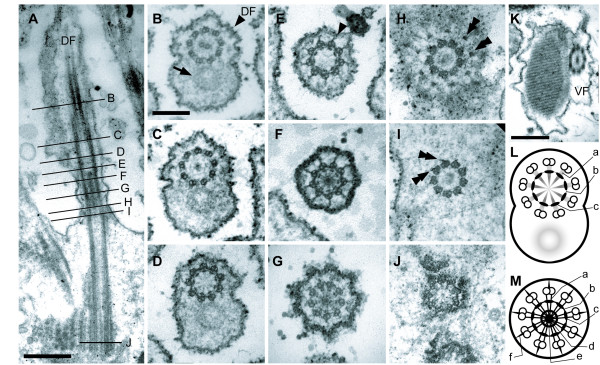
**Transmission electron micrographs (TEM) showing paraxonemal rods in the flagella, the flagellar transition zone and the basal bodies of *Calkinsia aureus***. **A**. Longitudinal section of the dorsal flagellum (DF) showing the flagellar transition zone and the dorsal basal body (DB) (bar = 500 nm).** B-J**. Non-consecutive serial sections through the DF (**B**), the flagellar transition zone (**C-G**), and the DB (**H-J**) as viewed from anterior end (images at same scale, bar = 200 nm). **B**. Section showing the 9+2 configuration of axonemal microtubules and the tubular paraxonemal rod (arrow) in the DF. **C**. Section showing termination of central microtubules and the 9+0 configuration of axonemal microtubules. **D**. Section showing the transition zone through an outer concentric ring associated with nine electron dense globules inside of each doublet and faint spokes that extend inward from the each globule (see **L **for a diagram of this micrograph). **E**. Section through the nine radial connectives (arrowhead) that extend outward from each doublet to the flagellar membrane. **F**. Section showing the radial connectives that extend outward toward the flagellar membrane, the spokes that extend inward from the microtubular doublets, the central electron dense hub, and inner concentric rings (see **M **for the diagram of this micrograph). **G**. Section showing the electron dense hub and inner and outer concentric rings, and the absence of radial connectives. **H**. A section at the level of the insertion of the DF. The transitional fibers (double arrowheads) extending from the microtubular triplets of the DB are shown. **I**. Section through the area just below the distal boundary of the DB. The transitional fibers (double arrowheads) connect to each microtubular triplet. **J**. Section through the proximal region of the DB showing the cartwheel structure. **K**. View through the paraxonemal rod of the ventral flagellum (VF) (bar = 500 nm). **L**. Diagram of the level of **D **showing faint spokes (a) that extend inward from each globule, an outer concentric ring (b) and nine electron dense globules (c). **M**. Diagram of the level of **F **showing spokes (a), an outer concentric ring (b), nine electron dense globules (c), an electron dense hub (d), an inner concentric ring (e) and radial connectives (f).

**Figure 7:**
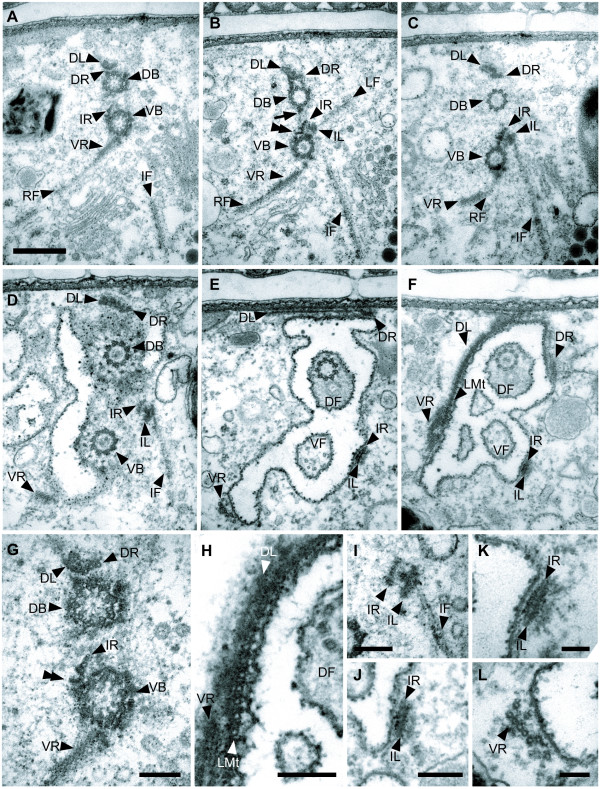
**Transmission electron micrographs (TEM) showing the organization of microtubular roots that originate from the dorsal and ventral basal bodies (DB and VB, respectively)**. Those are viewed from the anterior end (**A-F **at same scale, bar = 500 nm). **A**. The proximal region of the basal bodies close to the cartwheel structure. The dorsal root (DR) originates from the DB; the intermediate root (IR) and the ventral root (VR) extend from the VB. A dorsal lamina (DL) attaches to the dorsal side of the DR; the right fiber (RF) is close to the ventral side of the VR. **B**. Section showing the right fiber (RF), the IR-associated lamina (IL), a left fiber (LF) and an intermediate fiber (IF) associated with the VB. The arrow points to the connective fiber between the DB and the VB. Dense fibrils (double arrowhead) extend to the right side of each microtubule of the intermediate root (IR). **C**. Section through the middle part of the DB and the VB. **D**. Section through the insertion of the flagella. **E**. Section through the flagellar transition zone showing the extension of the DL and disorganization of the VF. **F**. Section showing the linked microtubules (LMt) associated with the dorsal lamina (DL) and the ventral root (VR). **G**. High magnification view of proximal area of the two basal bodies, the DB and the VB, of **A **showing the cartwheel structure and the dorsal lamina (DL) on the dorsal side of the dorsal root (DR). The double arrowhead indicates the fibril from each microtubule of the IR. **H**. High magnification view of right wall of the pocket of **F **showing the LMt and the DL. **I**. High magnification view of the IR of **D **showing the relationship among the IR, IL and IF. **J**. High magnification view of the IR at the level of **E **showing the IR and IL. **K**. High magnification view of the IR and IL. **L**. High magnification view of the VR in **E**. (**G-L**, bars = 200 nm).

**Figure 8:**
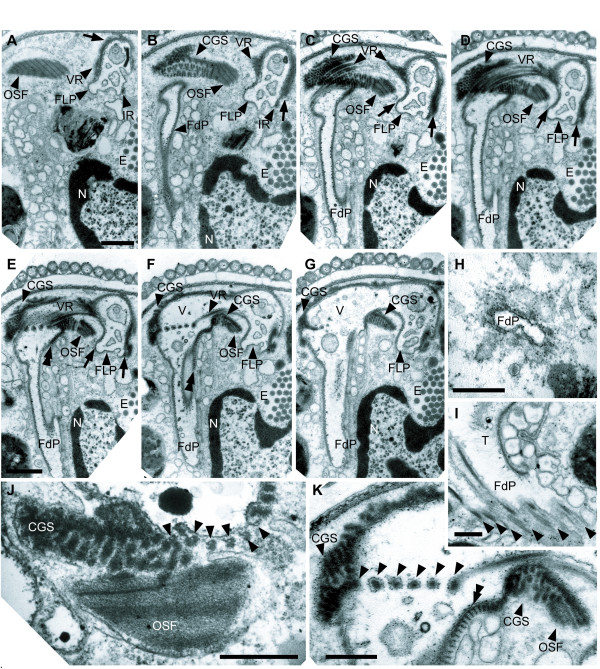
**Transmission electron micrographs (TEM) of *Calkinsia aureus *showing the feeding apparatus**. The ventral flagellum was disorganized in all sections (A-D at same scale, bar = 1 μm; E-G at same scale, bar = 1 μm). **A**. Section showing the oblique striated fibrous structure (OSF) and the VR along the wall of the flagellar pocket (FLP). Arrow points out the LMt and the DL. **B**. Section through the congregated globular structure (CGS), the OSF and the feeding pocket (FdP). The VR extends to the right. The arrow points out the LMt and the DL, which extend from the VR to the IR and support the dorsal half of the FLP. **C**. Section showing the VR over the CGS. Arrows show the LMt and DL. **D**. The VR crosses over the CGS and extends to right side of the FdP. Most of the wall of the FLP is supported by the LMt and DL (arrows). **E**. A striated fiber (double arrowhead) supports the left side of the FdP and extends from the left side of the CGS. Arrows indicate the extension of the LMt and DL. **F**. Section through the beginning of the vestibulum (V) and the striated fiber (double arrowhead). **G**. The V is enlarged and the CGS remains at both sides of the FdP. **H**. High magnification of FdP. **I**. Tangential TEM section showing the VR with an electron dense fiber along the feeding pocket and a tomentum (T) of fine hairs. **J**. Longitudinal section through the CGS and the OSF. Six ventral root microtubules embedded within the electron dense fibers (arrowheads). **K**. High magnification view of the VR supporting the FdP shown in **F**. Double arrowhead indicates the striated fiber and the six arrowheads indicate the electron dense fibers of the VR. (**H-K**, bars = 500 nm).

**Figure 9:**
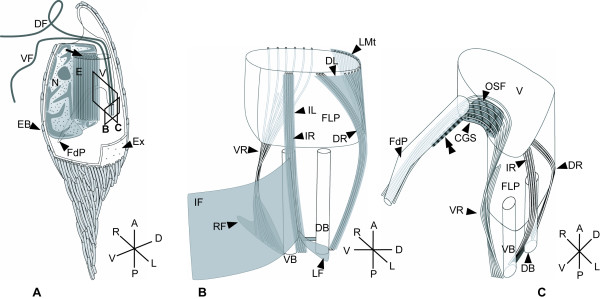
**Diagram of the cell (A), the flagellar apparatus (B) and the feeding apparatus (C) of *Calkinsia aureus *based on serial TEM sections**. **A**. Illustration of the cell viewed from the left side; arrow marks the extrusomal pocket. Boxes B and C indicate the plane of view shown in Figures B and C, respectively. **B**. Illustration of the flagellar apparatus as viewed from left side. **C**. Illustration of the feeding apparatus as viewed from anterior-ventral side. The double arrowhead marks the striated fiber along the feeding pocket (FdP). Note DL, IF, IL, LF, LMt, and RF are not shown on this diagram for clarity.

An anterior nucleus was positioned near the ventral side of the cell and contained a prominent nucleolus and condensed chromosomes (Figures 3A, 3C–D). The vestibulum was positioned directly above the nucleus as this space passed from the ventral, subapical opening toward the dorsal side of the cell (Figure 3C). The vestibulum then extended posteriorly along the dorsal side of the cell and branched into three distinct pockets: (1) a novel "extrusomal pocket", (2) a flagellar pocket and (3) a feeding pocket (Figures 3A, 3C; described in more detail below). A battery of longitudinally arranged extrusomes was connected to the base of the extrusomal pocket and was nested within a notch on the dorsal side of the ventral nucleus (Figures 1B, 3A, 3C). Each extrusome was about 160 nm in diam. (Figure 3G). The battery of extrusomes was indistinguishable from the feeding rods of euglenids when viewed with the light microscope, and discharged as a single unit through the anterior opening (Figures 1B, 1H). The flagellar pocket was located on the dorsal side of the cell and contained two flagella that inserted at the bottom of the pocket (Figures 6, 7; described in more detail below). The feeding pocket was located to the right of the flagellar pocket and extended horizontally before tapering posteriorly toward the ventral side of the cell (Figures 8, 9; described in more detail below).

### Flagella, Transition zones and Basal Bodies

Both flagella contained a paraxonemal rod adjacent to the axoneme, and flagellar hairs were not observed on either flagellum (Figure 6A). The paraxonemal rod in the dorsal flagellum (DF) had a whorled morphology in transverse section, and the paraxonemal rod in the ventral flagellum (VF) was constructed of a three-dimensional lattice of parallel fibers (Figures 6B, 6K). The entire length of the axoneme had the standard 9+2 architecture of microtubules (Figure 6B). The central microtubules within each axoneme terminated approximately 1 μm above the flagellar insertion (Figures 6A, 6C); this 9 + 0 arrangement is recognized as the transition zone in accordance with the definition proposed by Hibberd [19]. The transition zone and basal bodies are further described here from the distal end toward the proximal end.

The central space within the proximal half of the transition zone contained three distinct elements: faint spokes (denoted as 'a'), an outer concentric ring positioned just inside the microtubular doublets (denoted as 'b'), and electron dense globules (denoted as 'c') (Figures 6D, 6L). Each faint spoke extended from a microtubular doublet toward the center of the transition zone. The globules were positioned at the intersections of each faint spoke and the outer concentric ring (Figures 6D, 6L). In more proximal points along the transition zone, nine "radial connectives" extended from each doublet toward the flagellar membrane (Figures 6E–F), and an opaque core was present within the central space when observed in both longitudinal and transverse section (Figures 6A, 6F–G). The opaque core consisted of six distinct elements: nine spokes extending from each doublet (denoted as 'a'), the outer concentric ring (denoted as 'b'), nine electron dense globules associated with the outer concentric ring (denoted as 'c'), a central electron dense hub (denoted as 'd'), an inner concentric ring (denoted as 'e') and nine radial connectives extending from each doublet to the flagellar membrane (denoted as 'f') (Figures 6F, 6M). The radial connectives disappeared just above the distal boundary of the basal body (Figures 6A, 6G), and the elements within the central space disappeared just below the distal boundary of the basal body (Figures 6A, 6H).

The dorsal basal body (DB) and ventral basal body (VB) anchored the dorsal flagellum (DF) and ventral flagellum (VF), respectively. Both basal bodies were approximately 1.6 μm long, arranged in parallel to each other, and possessed nine transitional fibers extending from each triplet towards the cell membrane (Figures 6A, 6H–I). Internal cartwheel elements were present within the most proximal ends of both basal bodies (Figures 6J, 7G).

### Flagellar Root System

The flagellar root system is described here from the proximal boundary of the basal bodies toward the distal boundary of the basal bodies as viewed from the anterior end of the cell (Figure 7). The DB and the VB were joined with a connecting fiber and associated with three microtubular roots: the dorsal root (DR), the intermediate root (IR) and the ventral root (VR) (Figures 7A–B). The VB, IR and VR were also associated with three fibrous roots: the right fiber (RF), the intermediate fiber (IF) and the left fiber (LF) (Figure 7B). The DR and IR were associated with two thin laminae: the dorsal lamina (DL) and the IR-associated lamina (IL), respectively (Figures 7A–D, 9B).

The DR originated from the dorsal side of the DB and consisted of three microtubules near the proximal boundary. Both the DR and the DL extended toward the anterior side of the cell (Figures 7B–D) and supported the flagellar pocket (Figures 7E–F). The DR occupied the dorsal left side of the flagellar pocket; the DL occupied the dorsal right side of the flagellar pocket and extended from the VR to the DR at the level of the transition zone (Figures 7E–F). A row of linked microtubules (LMt) originated in close association with the DL (above the VR) and supported the right side of the flagellar pocket (Figures 7F, 7H). The DL and LMt extended from the left side of the flagellar pocket to the right side near the posterior boundary of the vestibulum (Figures 8A–E). The LMt supported the inner lining of the vestibulum, turned posteriorly along the curve formed by the ventral opening (Figure 3E) and ultimately became the sheet of microtubules located beneath the plasma membrane of the entire cell (Figures 4A, 4C–D).

The IR was positioned between the two basal bodies, originated from the right dorsal side of the VB, and consisted of four microtubules near the proximal boundary (Figures 7B–C, 7G). The left side of the IR was tightly associated with the IL and two fibrous roots: the LF and the IF (Figure 7B). The LF extended laterally and was about 500 nm long; the IF extended to the left ventral side of the cell and was about 1.5 μm long (Figures 7B–C). The IL was associated with the left side of the IR along its entire length, and the IR and IL became more closely associated as they extended anteriorly along the left side of the flagellar pocket (Figures 7I–K). The microtubules from the IR eventually merged with the left side of the LMt-DL and likely contributed to the sheet of microtubules located beneath the plasma membrane of the entire cell (Figures 8A–C).

The VR originated from the ventral side of the VB and consisted of nine microtubules that were closely associated with the RF (Figures 7A, 7G). The RF extended toward the right-ventral side of the cell and was about 1 μm long (Figures 7A–C). The microtubules from the VR supported the right side of the flagellar pocket and joined the right side of the LMt and the DL (Figures 7D–F, 7L). The microtubules from the VR ultimately became one of the elements that reinforced the feeding apparatus (Figures 8, 9).

### Feeding Apparatus

The feeding apparatus was positioned on the right side of the flagellar pocket and is described here along the posterior to anterior axis. This apparatus consisted of four main elements or spaces: a feeding pocket, a VR embedded within six electron-dense fibers, a compact "oblique striated fiber" (OSF) and a "congregated globule structure" (CGS) (Figures 8, 9C). The OSF was approximately 1.5 μm long, 800 nm wide and 500 nm high and was positioned between the feeding apparatus and the right side of the flagellar pocket (Figures 8A, J). The CGS attached to the anterior side of the OSF (Figures 8B–E, 8J). Six rows of electron dense fibers, each containing a microtubule from the VR, passed over the top of the OSF-CGS complex (Figures 8C–F, 8J–K). The VR and the six associated fibers reinforced the anterior-right side of the feeding pocket (Figures 8C–E). The left side of the feeding pocket was reinforced by a striated fiber that extended from the left side of the CGS (Figures 8E–F, 8K, 9C).

The feeding pocket was surrounded by an accumulation of small vesicles and branched from the vestibulum toward the ventral side of the cell before turning toward the posterior end of the cell (Figures 8A–D, 9C). Serial oblique sections through the feeding pocket did not demonstrate distinctive feeding vanes or rods per se; only the VR microtubules within the electron dense fibers were observed (Figure 8H). Nonetheless, the vestibular junction (or crest) between the flagellar pocket and the feeding pocket contained a "tomentum" [20] of fine hairs (Figure 8I).

### Molecular Phylogenetic Position as Inferred from SSU rDNA

**Figure 10:**
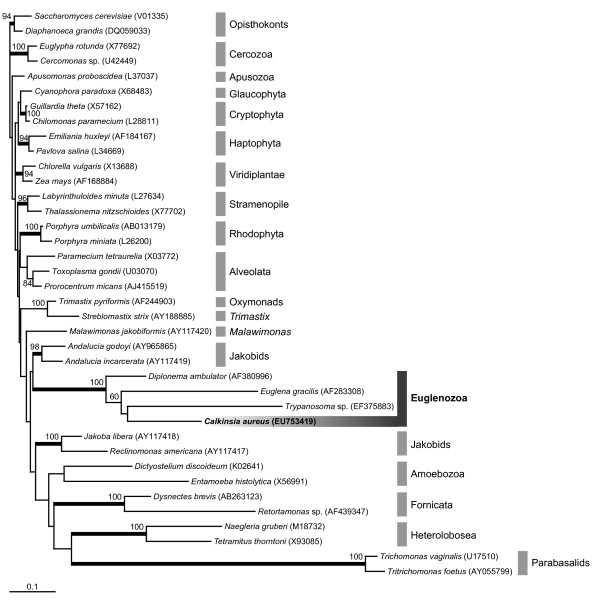
**Phylogenetic position of *Calkinsia aureus *within eukaryotes as inferred from SSU rRNA gene sequences**. Maximum likelihood (ML) analysis of 38 taxa sampled from phylogenetically diverse eukaryotes. This tree is rooted with opisthokont sequences. ML bootstrap values greater than 50% are shown. Thick branches indicate Bayesian posterior probabilities over 0.95. GenBank accession numbers of the sequences analyzed are shown in parentheses.

**Figure 11:**
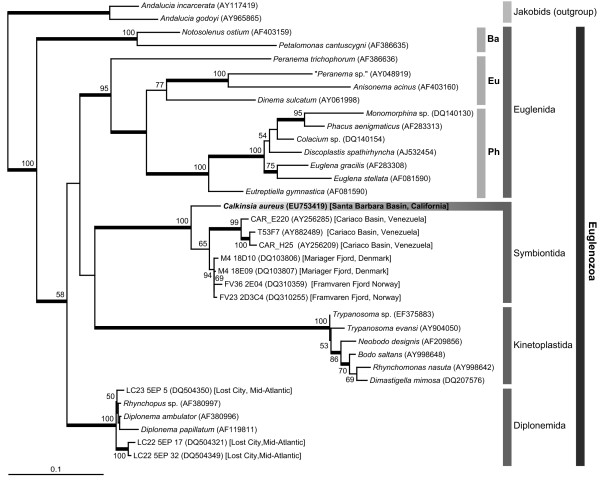
**Phylogenetic position of *Calkinsia aureus *within euglenozoans as inferred from SSU rRNA gene sequences**. Maximum likelihood (ML) analysis of 35 taxa focusing on the position of *Calkinsia aureus *within the Euglenozoa clade. Two jakobids, *Andalucia incarcerata *and *A. godoyi*, are used as outgroups in this analysis. ML bootstrap values greater than 50% are shown. Thick branches indicate Bayesian posterior probabilities over 0.95. Ba, bacteriotroph; Eu, eukaryotroph; Ph, phototroph. GenBank accession numbers of the sequences analyzed are shown in parentheses.

We determined the nearly complete sequence of the SSU rRNA gene of *C. aureus *(2034 bp). Maximum likelihood (ML) analyses of (i) a 38-taxon alignment including representative sequences from the major lineages of eukaryotes, robustly grouped the sequence from *C. aureus *with the Euglenozoa (e.g. *Euglena*, *Diplonema *and *Trypanosoma*) (Figure 10). In order to  more comprehensively evaluate the phylogenetic position of *C. aureus* within the Euglenozoa, we analyzed three additional datasets: (ii) a 35-taxon alignment (Figure 11), (iii) a 29-taxon alignment (Additional file 1), and (iv) a 25-taxon alignment (Addtional file 2) (see Methods for details).

Tree topologies of these three ML analyses were very similar (Figure 11, Additional Files 1, 2). Accordingly, the results from the analyses of the 35-taxon dataset including several short environmental sequences, was an accurate representation of all three analyses (Figure 11). Euglenozoan sequences clustered into five major groups with high statistical support: a kinetoplastid clade, a diplonemid clade, a bacteriovorous euglenid clade (i.e. *Notosolenus *and *Petalomonas*), a clade consisting of eukaryovorous and photosynthetic euglenids, and a novel clade referred to here as the "Symbiontida". The relationships among these clades (i.e. the backbone) were not resolved (Figure 11). Additional phylogenetic analyses using alternative outgroups (e.g., heteroloboseans) recovered the same basic tree topology shown in Figure 11: (1) *Calkinsia aureus *is a member of a distinct euglenozoan subclade consisting of sequences derived from environmental PCR surveys, and (2) this clade is not convincingly affiliated with any one of the three known euglenozoan subgroups (euglenids, kinetoplastids and diplonemids). Moreover, the sequence from *C. aureus *occupied the deepest position within the Symbiontida, which otherwise consisted of seven environmental sequences collected from Northern Europe and South America (Figure 11).

## Discussion

Several poorly studied flagellates, some with discoidal-shaped mitochondrial cristae, have, at one time or another, been suspected to be close relatives of euglenozoans (e.g. *Stephanopogon*, *Hemimastix*, *Bordnamonas, Cryptaulax, Postgaardi *and *Calkinsia*) [21-24]. The best synapomophies for the Euglenozoa are (1) a tripartite flagellar root system (DR, IR and VR), (2) heteromorphic paraxonemal rods (i.e. a whorled structure in the DF and three-dimensional lattice of parallel fibers in the VF), and (3) tubular extrusomes [9]. The presence of these ultrastructural features in very diverse lineages of flagellates, in combination with molecular phylogenetic data, has established the identity and composition of the Euglenozoa [7,9].

*Calkinsia aureus *was originally described as a member of the Euglenida with light microscopical information [12], and we demonstrate here that these flagellates possess all three ultrastructural synapomorphies for the Euglenozoa. Moreover, the permanently condensed chromatin, long flagellar transition zone, longitudinal cell division and long basal bodies are also features found in many other euglenozoans [25]. These morphological data were concordant with our comparative analyses of SSU rDNA showing that *C. aureus *is robustly embedded within the Euglenozoa clade (Figures 10, 11). However, *C. aureus *lacked traits that are specific to any of the three previously recognized euglenozoan subgroups (e.g., kinetoplasts, pellicle strips, or absence of paraxonemal rods). The faintly striated pellicle originally attributed to *C. aureus *using light microscopy is, in actuality, the longitudinally arranged rod-shaped epibiotic bacteria [13,14]. The sheet of microtubules beneath the plasma membrane in *C. aureus *was continuous over the entire cell, like in kinetoplastids and diplonemids, rather than interrupted by periodic discontinuities like in euglenids [26-28] (Figure 3C). There was also no clear evidence of a euglenid-like feeding apparatus consisting of rods and vanes [20,26,29]. Accordingly, our study indicates that with present knowledge, *C. aureus *should not be considered a member of the Euglenida or more specifically, a member of the Petalomonadidae as originally classified [12].

### Absence of Mitochondria with Cristae

Aerobic kinetoplastids and euglenids possess well-developed discoid-shaped cristae within their mitochondria [26], and diplonemids and *Hemistasia *possess a few flat-shaped cristae within each mitochondrion [30-32]. By contrast, both *C. aureus *and *P. mariagerensis *lack recognizable mitochondria with cristae, and instead, contain double-membrane bound organelles that are nearly identical in morphology to the well-studied hydrogenosomes described in other anoxic flagellates (e.g. *Trichomonas*) [33]. Hydrogenosomes are the descendents of mitochondria and function to produce molecular hydrogen, acetate, CO_2 _and ATP in anoxic environments [34,35]. A more confident functional characterization of the mitochondrion-derived organelles in *C. aureus *or *Postgaardi *will require biochemical and molecular biological assays.

### A Novel Extracellular Matrix

The plasma membrane of *C. aureus *was reinforced with a continuous sheet of microtubules and a double-layered lamella, which was in turn subtended by a dense array of mitochondrion-derived organelles (Figures 4, 5). This overall organization, where mitochondrion-derived organelles are located immediately beneath a sheet of surface microtubules, has also been observed in *Postgaardi*. However, a uniform and perforated extracellular matrix enveloped the cell surface of *C. aureus*, and so far as we know, the organization of this cell covering is novel not only among euglenozoans, but also among eukaryotes (Figures 4, 5). Because both the epibiotic bacteria and the host cell cytoplasm were colorless (Figures 1D, 1F–G), the distinctively orange color of *C. aureus *is clearly attributable to the chemical composition of the extracellular matrix (Figure 1G). Moreover, the even distribution of tiny tubes within the matrix provide conduits between the host plasma membrane and the epibiotic bacteria and presumably facilitate metabolic exchanges necessary for survival in low-oxygen environments. This interpretation is consistent with knowledge of anoxic ciliates, which also maintain an intimate physical relationship between mitochondrion-derived organelles (immediately beneath the host plasma membrane) and epibiotic bacteria (immediately above the host plasma membrane) [36,37].

### Flagellar Apparatus

The flagella of most euglenids and kinetoplastids have non-tubular mastigonemes (or flagellar hairs) that, among other functions, facilitate gliding motility [38]; however, these structures are absent in *C. aureus*, *P. mariagerensis *and diplonemids. Instead, a tomentum of fine hairs are present at the crest of the feeding pocket in *C. aureus *that are similar to those described in the phototrophic euglenid *Colacium *[39], the phagotrophic euglenid *Peranema *[40], and the kinetoplastid *Cryptobia *[41,42]. Although hairs associated with the feeding pocket have not been routinely observed in other members of the Euglenozoa, the presence of these hairs in distantly related lineages indicate that this feature might represent an ancestral state for the Euglenozoa as a whole.

The structure of the flagellar transition zone is variable among kinetoplastids and euglenids, particularly in regard to the presence/absence of peripheral elements and transitional plates. Kinetoplastids and diplonemids possess distal and proximal transitional plates and a hollow transition zone [30,32,42], while euglenids only possess the proximal transitional plate. Although the transition zone of most euglenids is also hollow, the transition zone in some euglenids, such as *Entosiphon applanatum *and *Notosolenus *(*Petalomonas*)*mediocanellata*, has been shown to be electron dense. However, the detailed structure of these transition zones still remains to be characterized in detail [29,43]. The central area of the transition zone in *C. aureus *is also electron dense and contains a complex system of elements that have never been observed in any other Euglenozoan so far (Figure 6). Characterization of the flagellar transition zone in *Postgaardi *might demonstrate several homologous elements that would help to further establish a close relationship between this lineage and *C. aureus*.

Nonetheless, *Diplonema ambulator, Rhynchopus euleeides*, *R. coscinodiscivorus *and *C. aureus *all have fibers that extend from each microtubular doublet to the flagellar membrane; these fibers have been called "transitional fibers" [30,32,44]. "Transitional fibers" has also been used to describe fibers that extended from each microtubular triplet of a basal body to the flagellar membrane, which is potentially confusing [45-47]. Nonetheless, the "radial connectives" extending from the doublets in the transition zone of *C. aureus *are nearly identical, and likely homologous, to the 'transitional fibers' extending from the doublets in diplonemids, such as *D. ambulator*.

### Feeding Apparatus

Each of the euglenozoan subgroups contains members with an elaborate feeding apparatus [20,26,29,39]. Most phagotrophic euglenids, for instance, have a distinctive feeding apparatus consisting of 4–5 central vanes and 2–3 supporting rods [28,48,49]. Some bacteriovorous euglenids (e.g. *Petalomonas*), however, possess a much simpler feeding apparatus that is very similar to the MTR feeding pockets found in many kinetoplastids (e.g. *Bodo*) [26]. The microtubules that support the rods in phagotrophic euglenids and the MTR pockets in bacteriovorous euglenozoans originate from the ventral root of the ventral basal body. Similarly, the feeding pocket in *C. aureus *was also supported by microtubules that originated from the ventral root and is almost certainly homologous to the MTR pockets or rods found in other euglenozoans, including *Postgaardi *[33]. Moreover, the compact "oblique striated fiber" (OSF) that reinforces the feeding pocket in *C. aureus *was similar to the amorphous matrix found in some euglenid feeding rods and might represent a vestige of a more elaborate ancestral state. However, this inference will require improved understanding of the morphological diversity and phylogeny of other euglenozoans that are more closely related to *C. aureus*.

### A Novel Extrusomal Pocket

Although tubular extrusomes are not widespread within the Euglenozoa, several members from each main subgroup possess them, such as the euglenid *Entosiphon *[50,51]; the kinetoplastids *Rhynchobodo *[52], *Hemistasia *[31], and *Phylomitus *[53]; the diplonemid *Diplonema nigricans *[54]; and *Postgaardi mariagerensis *[33]. *Calkinsia aureus *not only had tubular extrusomes like the lineages listed above, but they were clustered together much like the single battery of tubular extrusomes found in *Hemistasia *[31]. By contrast, *Postgaardi *and *Rhynchobodo *possess several smaller batteries of tubular extrusomes that are dispersed throughout the cytoplasm [33,52].

The battery of tubular extrusomes in *C. aureus *was anchored to a novel extrusomal pocket that branched off of the vestibulum separately from the feeding apparatus and the flagellar apparatus (Figures 3A, 3C, 9). This battery of extrusomes was often discharged as a single unit from the extrusomal pocket and through the anterior opening (Figure 1H). The functional significance of this process is unclear.

The phagotrophic euglenid *Dinema sulcatum *also contains a flagellar pocket and reportedly has two additional pockets: (1) a "normal" feeding apparatus consisting of supportive rods and vanes and (2) an "extra" pocket consisting of MTR-like microtubules [43]. One previously proposed hypothesis for the presence of two feeding pockets in *D. sulcatum *involves the following inferences: the "extra" pocket is a remnant of the MTR feeding pocket present in the ancestral euglenozoan and the rod-and-vane based feeding apparatus represents a duplicated, and greatly embellished, MTR pocket that arose within a derived lineage of phagotrophic euglenids [7,27,55]. This hypothesis is consistent with comparative morphological data that indicates other euglenid cytoskeletal components also evolved by duplication, such as the total number of pellicle strips around the cell periphery [7,28,56,57]. Nonetheless, the extrusomal pocket in *C. aureus *was supported by the LMt (connected to the dorsal root) rather than microtubules from the ventral root, which support both MTR pockets and rod-and-vane based feeding apparatuses in euglenozoans. Therefore, the extrusomal pocket in *C. aureus *appears to be novel and does not seem to be homologous to any type of feeding apparatus reported so far (e.g. a rod-and-vane based apparatuses or a remnant or duplicated MTR pocket).

### Euglenozoans with Epibiotic Bacteria

*Postgaardi mariagerensis *[33,58], *Euglena helicoideus *[59], *Dylakosoma pelophilum *[60], *C. aureus *[13] and five unidentified euglenozoans from low oxygen environments [13,14,61] have been reported to possess epibiotic bacteria on the cell surface. The epibiotic bacteria on *D. pelophilum *are spherical, and those on the other taxa are rod-shaped and densely packed on the cell surface. Only one of the five unidentified euglenozoans, namely "morphotype C" from Monterey Bay, was studied with both SEM and TEM [61]. The rod-shape epibiotic bacteria on these cells were not associated with a superficial distribution of mitochondrion-derived organelles (e.g., hydrogenosomes) beneath the host plasma membrane. Nonetheless, morphotype C was clearly a euglenid, because the flagella contained paraxonemal rods, the feeding apparatus consisted of rods and vanes, and thin proteinaceous strips supported the cell surface.

By contrast, the combination of ultrastructural features in *C. aureus *and *P. mariagerensis *make these lineages difficult to place within the Euglenozoa. Both lineages lack evidence of pellicle strips or kinetoplasts and possess paraxonemal rods, tubular extrusomes, mitochondrion-derived organelles beneath the plasma membrane, and condensed chromatin. Detailed comparisons of the feeding apparatus in *C. aureus, P. mariagerensis*, and other anoxic euglenozoans should help better establish their phylogenetic relationships with each other; however, except for *C. aureus*, this information is currently lacking for nearly all of these lineages, including *P. mariagerensis*.

### Molecular Phylogenetic Framework for Euglenozoans in Low-Oxygen Environments

The morphology of *C. aureus *(e.g. the flagellar apparatus and tubular extrusomes) was completely concordant with the molecular phylogenetic data in so far as strongly placing *C. aureus *within the Euglenozoa, but not with any of the three previously recognized subclades. Figure 11 shows the phylogenetic position of *C. aureus *within the Euglenozoa, which consisted of five main clades. Although *Petalomonas *and *Notosolenus *branched together as a separate clade, morphological evidence strongly supports their inclusion within the Euglenida. Therefore, the molecular phylogenetic data coupled with the morphological data allows us to recognize four clades of euglenozoans: the Euglenida, the Kinetoplastida, the Diplonemida and a novel clade of anoxic euglenozoans, hereby named the Symbiontida.

The Symbiontida includes several environmental sequences that were originally designated either as diplonemid sequences (e.g. T53F7) [62], as uncultured euglenozoan sequences (e.g. M4 18E09, M4 18D10, FV23 2D3C4 and FV36 2E04) [63,64] or as "possible early branching eukaryotes" (CAR_H25 and CAR_E220) [65]. Some of the environmental sequences within the Symbiontida were already suspected to represent either a novel sister clade to the Euglenozoa or novel subclade of euglenozoans [64]. Nonetheless, we have demonstrated that the Symbiontida contains several more environmental sequences collected from different low-oxygen environments and also *C. aureus*, which provides an organismal anchor (i.e. the cellular identity) for this clade.

We should also note that some environmental sequences from mid-Atlantic hydrothermal vent environments in the "Lost City", namely LC23 5EP 5, LC22 5EP 17, and LC22 5EP 32, grouped strongly with the diplonemid clade and not with the Symbiotida [66]. Moreover, the lack of phylogenetic signal and perhaps also long-branch-attraction were the likely reasons for why the relatively fast-evolving sequences from *Notosolenus *and *Petalomonas *did not cluster strongly with the euglenid clade in our analyses of the dataset containing the shortest sequences (Figure 11). Our analysis of the dataset including only the longest sequences, by contrast, clustered *Notosolemus *and *Petalomonas *with all other euglenids, albeit without strong statistical support (Additional File 2) [67,68].

### The Symbiontida: A Novel Subclade of the Euglenozoa

Before *C. aureus *had been studied at the ultrastructural and molecular phylogenetic levels, one author classified this lineage with *P. mariagerensis *within the taxon "Postgaardea" on the basis of microaerophily [10,11]. Although our characterization of *C. aureus *has demonstrated epibiotic bacteria and mitochondrion-derived organelles like those described in *P. mariagerensis*, the presence of these characters in both lineages does not necessarily reflect homology. Independently derived physical relationships between epibiotic bacteria and mitochondrion-derived organelles have been found in many different lineages of anoxic microeukaryotes, such as ciliates, oxymonads, parabasalids, heteroloboseans and euglenozoans [36,69]. Moreover, the presence of tubular extrusomes in both *C. aureus *and *P. mariagerensis *could be a symplesiomorphic state inherited from a very distant euglenozoan ancestor.

Nonetheless, our phylogenetic analyses demonstrate that *C. aureus *is a member of a newly recognized clade of anoxic euglenozoans consisting mainly of environmental sequences. The absence of molecular phylogenetic data and conclusive ultrastructural data from *Postgaardi *precludes us from determining whether this lineage is also a member of the clade of microaerophiles. Until these data are reported and the phylogenetic position of *Postgaardi *is demonstrated more rigorously, we concur with a previous taxonomic treatment for *Postgaardi *that recognizes this lineage as incertae sedis within the Euglenozoa [3]. As such, we conclude that it is premature to recognize the taxon Postgaardea and view it as a synonym for *P. mariagerensis*.

In light of the previous discussion, we propose the name "Symbiontida" for the clade of microaerobic or anaerobic euglenozoans consisting of the most recent ancestor of *C. aureus *that also possessed rod-shaped epibiotic bacteria, reduced or absent mitochondrial cristae, tubular extrusomes and a nucleus with permanently condensed chromatin. This novel subclade of euglenozoans is recognized on the basis of SSU rDNA-based phylogenetic data of lineages from several different low-oxygen environments. Although the ultrastructural characteristics listed above are expected to be present in most, if not all, members of the Symbiontida (the ultrastructural and molecular phylogeny of another lineage in this clade will be published shortly; Breglia, Yubuki, Hoppenrath and Leander, in preparation), this remains to be demonstrated with improved knowledge of euglenozoan diversity from both ultrastructural and molecular phylogenetic perspectives.

### Phylogenetic (apomorphy-based) diagnosis

Euglenozoa Cavalier-Smith 1981

Symbiontida taxon nov. Yubuki, Edgcomb, Bernhard & Leander, 2009

#### Apomorphy

Rod-shaped epibiotic bacteria above superficial layer of mitochondrion-derived organelles with reduced or absent cristae, homologous to the organization in *Calkinsia aureus*, the type species (Figures 2, 4).

### Extended diagnosis of the type species

*Calkinsia aureus *Lackey, 1960, emend., Yubuki, Edgcomb, Bernhard & Leander, 2009

Paraxonemal rods present in flagella; kinetoplast DNA and pellicle strips absent; long complex transitional zone between the basal bodies and the axonemes. Rod-shaped epibiotic bacteria on perforated orange extracellular matrix. Cell with a large nucleus on the anterior ventral side and a battery of tubular extrusomes linked to an extrusomal pocket located adjacent to the nucleus. Feeding apparatus supported by both fibrous structures and microtubules that are derived from ventral root (VR). Small subunit ribosomal RNA gene sequence (EU753419) distinguishes *Calkinsia aureus *from all other symbiontid species.

## Conclusion

Molecular phylogenies inferred from SSU rDNA demonstrate that *C. aureus *is closely related to several marine environmental sequences collected from low-oxygen environments, forming a novel subgroup within the Euglenozoa, referred to here as the "Symbiontida". Improved understanding of these flagellates is necessary for further demonstrating the cellular identity of the Symbiontida and for reconstructing the evolutionary radiation of the euglenozoan lineage. In this study, we characterized the detailed ultrastructure of *C. aureus *and demonstrated all of the euglenozoan synapomorphies (e.g. flagellar apparatus) and several cellular innovations associated with symbiotic interactions with epibiotic bacteria (e.g., complex extracellular matrix). We also demonstrated novel ultrastructural systems found in this species, such as the extrusomal pocket.

Environmental sequencing surveys from different low-oxygen environments around the world suggest that many symbiontid lineages have yet to be discovered and characterized. Continued exploration into the overall diversity of this group should contribute significantly to our understanding of eukaryotic evolution, especially in low-oxygen environments.

## Abbreviations

AF: anterior flagellum; B: epibiotic bacteria; Ba: bacteriotroph; CGS: congregated globule structure; DB: dorsal basal body; DF: dorsal flagellum; DL: dorsal lamina; DR: dorsal root; E: extrusome(s); EP: extrusomal pocket; Eu: eukaryotroph; Ex: extracellular matrix; G: Golgi body; GL: glycocalyx; IF: intermediate fiber; IL: IR-associated lamina; IR: intermediate root; LF: left fiber; LM: light microscope; LMt: linked microtubules; MtD: mitochondrion-derived organelle; N: nucleus; Nu: nucleolus; Os: osmotroph; OSF: oblique striated fibrous structure; PF: posterior flagellum; Ph: phototroph; RF: right fiber; S: somatonema; SBB: Santa Barbara Basin; SCB: sodium cacodylate buffer; SEM: scanning electron microscope; VB: ventral basal body; T: tomentum; TEM: transmission electron microscope; VF: ventral flagellum; VR: ventral root.

## Authors' contributions

NY carried out all of the LM, SEM, TEM and molecular phylogenetic work, wrote the first draft of the paper and participated in the collection of sediment samples from the SBB. VPE and JMB, the Chief Scientist, coordinated and funded the research cruise to the SBB. BSL funded and supervised the collection and interpretation of the ultrastructural and molecular phylogenetic data and contributed to writing the paper. All authors have read, edited, and approved the final manuscript.

## Supplementary Material

Additional file 1 **Maximum likelihood (ML) analysis of 29 taxa focusing on the position of *Calkinsia aureus *within the Euglenozoa clade.** Two jakobids, *Andalucia incarcerata *and *A. godoyi*, are used as outgroups in this analysis. The short environmental sequences are excluded from the dataset used in Figure 11 and fast-evolve euglenids sequences, *Ploeotia, Menoidium *and* Astasia*, are included. ML bootstrap values greater than 50% are shown. Thick branches indicate Bayesian posterior probabilities over 0.95. Ba, bacteriotroph; Eu, eukaryotroph; Os, osmotroph; Ph, phototroph. GenBank accession numbers of the sequences analyzed are shown in parentheses. Click here for file

Additional file 2 **Maximum likelihood (ML) analysis of 25 taxa focusing on the position of *Calkinsia aureus *within the Euglenozoa clade.** Two jakobids, *Andalucia incarcerata *and *A. godoyi*, are used as outgroups in this analysis. The short environmental sequences are removed from the dataset used in Figure 11 and fast-evolve euglenids sequences, *Dinema*, *Ploeotia, Menoidium *and* Astasia*, are excluded. ML bootstrap values greater than 50% are shown. Thick branches indicate Bayesian posterior probabilities over 0.95. Ba, bacteriotroph; Eu, eukaryotroph; Ph, phototroph. GenBank accession numbers of the sequences analyzed are shown in parentheses. Click here for file
